# Exploring the willingness to pay for HPV vaccines and price sensitivity among Chinese college students: the impact of health literacy and vaccine hesitancy

**DOI:** 10.3389/fpubh.2025.1621416

**Published:** 2025-08-29

**Authors:** Yuan Li, Hiromi Kawasaki, Zhengai Cui, Sae Nakaoka, Yingai Cui, Md Moshiur Rahman

**Affiliations:** ^1^Graduate School of Biomedical and Health Sciences, Hiroshima University, Hiroshima, Japan; ^2^School of Humanities and Management, Guangdong Medical University, Dongguan, China; ^3^School of Nursing, Guangdong Medical University, Dongguan, China

**Keywords:** HPV vaccine, willingness to pay, health literacy, vaccine hesitancy, van Westendorp PSM, college students

## Abstract

**Background:**

Human papillomavirus (HPV) infection constitutes a substantial public health challenge in China. Despite proven vaccine efficacy, coverage remains critically low among high-risk sexually active college students. Out-of-pocket payment requirements contribute to the intention–behavior gap, while health literacy (HL) and vaccine hesitancy (VH) influence payment decisions. This study aims to directly quantify Chinese college students’ willingness to pay (WTP) and price sensitivity, examining HL and VH as key determinants.

**Methods:**

A convenience sample of 4,928 students at Guangdong Medical University in Dongguan, China was selected to complete a cross-sectional online survey (June 16–July 16, 2024) on the “Wenjuanxing” platform. We assessed socioeconomic status, HPV knowledge, HL, VH, and WTP through the survey and applied the van Westendorp price sensitivity meter (PSM) to quantify HPV vaccine price sensitivity.

**Results:**

A total of 67.6% of the participants expressed a WTP for an out-of-pocket HPV vaccine, with 87.2% preferring nine-valent vaccines. Key factors identified included monthly expenses, medical insurance, HPV knowledge, HL, and the VH items’ perceived necessity, importance, and vaccine safety. The van Westendorp PSM indicated that the market prices of the nine-valent vaccines exceeded the college students’ acceptable price ranges.

**Conclusion:**

Examination of HL and VH provided a valuable framework for understanding the WTP for HPV vaccines. The van Westendorp PSM confirmed that the price sensitivity of the nine-valent HPV vaccine exceeded its affordability. Integrating vaccines into medical insurance, implementing targeted tiered subsidies (e.g., an optimal subsidy of 269.81 CNY, or at least 126.20 CNY), and conducting tailored education addressing HL/VH should be prioritized as potential solutions.

## 1 Introduction

Human papillomavirus (HPV) infection constitutes a substantial and widespread public health challenge in China and globally (1, 2). Large-scale studies have demonstrated significant HPV infection rates among Chinese women, with the weighted high-risk HPV (HR-HPV) prevalence reaching 12.30% among women ≥20 years and an overall HR-HPV prevalence of 13.12% in cervical screening populations (2, 3). Persistent HR-HPV infection serves as the primary cause of cervical cancer, with approximately 90% of cervical cancer cases linked to high-risk HPV infection (4). Research indicates concerning HPV infection rates among Chinese men [e.g., 52.45% in male outpatients, 7.89% in healthy male examinees (5), and over 40% in Guangdong (6)].

Although HPV vaccination has proven efficacy in preventing HPV-related cancers (e.g., cervical, anogenital, head and neck cancers) (1), vaccination rates in China remain substantially low. The highest first-dose HPV vaccination rate in China reached only 14.02% among young adults aged 20–24 years, significantly below the global coverage rate of 20% for the first dose in 2019 (7). This limited coverage presents particular concerns among sexually active college students (18–25 years), a high-risk population where the mean age of sexual debut (18.39 years) aligns with peak HPV exposure risk for unvaccinated individuals (8, 9). Current data indicate only an 11% vaccination rate among Chinese female students (10), with comparable data for males (including male college students) remaining unavailable. Although multiple surveys have demonstrated that college students, particularly females (with rates exceeding 80%), are highly willing to receive HPV vaccination (11, 12), a substantial gap exists between intention and uptake, leaving this high-risk group vulnerable to HPV infection. While existing studies in China have primarily examined factors influencing vaccination willingness (10, 12), the key barriers preventing the conversion of this willingness into actual vaccination behavior remain insufficiently explored.

Behavioral decision theory suggests that converting vaccination willingness into action necessitates surpassing the critical willingness-to-pay (WTP) threshold, particularly for Chinese college students who pay for HPV vaccinations on their own while facing financial constraints (13). Health literacy (HL) serves as a fundamental factor in an individual’s ability to make informed health decisions (14). In conjunction with vaccine hesitancy (VH), HL influences the WTP for vaccines (15). Individuals with limited HL demonstrate increased vulnerability to vaccine-related misinformation (16), contributing to VH and consequently reducing WTP (17). Empirical studies have shown that when vaccine prices exceed affordability thresholds or reasonable price ranges, the actual vaccination rates and the WTP tend to be low, even amid high vaccination willingness (18, 19).

Despite WTP being critical as a behavioral bottleneck in vaccination uptake, comprehensive investigations into its determinants—particularly HL and VH—remain insufficient. Current studies demonstrate notable limitations: they primarily emphasize vaccination willingness rather than the mechanisms facilitating actual behavioral conversion and fail to comprehensively examine how HL and VH jointly affect WTP and price sensitivity thresholds. Additionally, evidence-based, specific subsidy policy recommendations, particularly for key college student populations, are lacking. This study therefore, aims to directly quantify Chinese college students’ WTP for HPV vaccines and their price sensitivity, specifically examining the mechanisms through which HL and VH influence these payment decisions. The findings provide essential evidence to guide the development of targeted and differentiated subsidy policies, ultimately reducing the gap between vaccination intent and behavior to increase uptake in this high-risk group.

## 2 Materials and methods

### 2.1 Survey design and study participants

This study was conducted from June 16 to July 16, 2024, at Guangdong Medical University in Dongguan, China. This study employed a convenience sampling method to conduct a cross-sectional survey on the Chinese online survey platform “Wenjuanxing”.^1^ The questionnaire was distributed to full-time students through WeChat. The inclusion criteria were as follows: (1) full-time enrolled students; (2) completion and successful submission of all mandatory questionnaire items, excluding questions skipped due to branching logic. The exclusion criteria were as follows: (1) a response time of less than 180 s; (2) inconsistencies in and illogical responses to some answers; and (3) failure to provide accurate academic and professional information.

The sample size was calculated *a priori* using the following formula on the basis of an error *α* of 0.05 and a permissible error *δ* of 0.05:


$$n=\frac{Z_{1-\alpha /2}^{2}*p(1-p)}{\delta^{2}}$$

Where *n* represents the sample size required for the survey, $Z_{1-\alpha /2}^{2}$ is the standard normal deviation of *α*, and *p* denotes the expected prevalence or positive rate of the survey, specifically referring to the HPV vaccination rates among Chinese college students. In this study, the proportion *p* was conservatively set at 0.5 to ensure the calculation of the largest minimum sample size. To account for the potential impact of invalid questionnaires, the sample size for this study was conservatively increased by 20%. Consequently, the final minimum sample size was 461 male and 461 female college students, resulting in a total minimum sample size of 922 participants.

A total of 5,384 individuals participated in the survey. After 456 responses (199 due to completion time <180 s; 45 for logical inconsistencies; 212 for unverifiable academic/professional information) were excluded, 4,928 valid questionnaires were retained (validity rate: 91.53%). Among these, 3,297 were female and 1,631 were male college students.

The study was conducted in accordance with the Declaration of Helsinki and approved by the Clinical Research Ethics Committee of the Affiliated Hospital of Guangdong Medical University (Approval No. PJKT2023-126). The study participants read and signed an online informed consent form. Potential study participants were informed that participation in the study was voluntary and that they could withdraw their decision to participate at any time.

### 2.2 Variables and measurements

#### 2.2.1 Sociodemographic characteristics

Eleven characteristics were assessed in this survey: sex, age, education level, household registration, college major, average monthly consumption, participation in government medical insurance, family monthly income, awareness of HPV, HPV vaccination status, and willingness to receive an HPV vaccine.

#### 2.2.2 HPV knowledge questionnaire

HPV knowledge was assessed via an 11-item questionnaire developed by Luo et al. (20). The total score ranged from 2 to 20 points, with a higher score reflecting a greater understanding of HPV. In this study, a passing score for HPV knowledge assessment was set to 12.8, corresponding to the 60th percentile.

#### 2.2.3 eHealth literacy scale

Health literacy was assessed using the Chinese version of the eight-item eHealth literacy (eHL) scale developed by Dong et al. (21). Each item was rated on a 5-point Likert scale, with 1 representing “strongly disagree” and 5 representing “strongly agree.” The total eHL score ranged from 8 to 40 points, with higher scores indicating greater levels of eHL. The Cronbach’s *α* of the scale was 0.944.

#### 2.2.4 Vaccine hesitancy scale

The vaccine hesitancy scale (VHS) utilized in this study was derived primarily from the HPV vaccine decision scale developed by Wei et al. (22). This scale included 12 items across 3 dimensions: necessity, importance, and safety. Each item was scored on a 5-point Likert scale, where a score of 1 indicated “strongly disagree” and a score of 5 indicated “strongly agree.” Notably, items within the necessity and safety dimensions were reverse scored; that is, a higher score reflected greater disapproval regarding the need for and safety of vaccination. In this study, the Cronbach’s *α* values for the necessity (consisting of 4 items), importance (consisting of 5 items), and safety (consisting of 3 items) dimensions of the VHS were 0.926, 0.885, and 0.852, respectively.

#### 2.2.5 HPV vaccine pricing questionnaire

The HPV vaccine pricing questionnaire comprised four sequential phases. First, participants who were willing to pay for HPV vaccines out-of-pocket selected their preferred vaccine type [quadrivalent vaccine (imported), nine-valent vaccine (imported), or other alternatives] on the basis of the provided product information.

Second, participants were required to provide 4 key pricing judgments regarding the selected vaccines by answering the following questions (23): (1) What price would make you doubt the quality of the vaccine and thus not buy it (too cheap)? (2) At what price would you consider this product to be of good value (cheap)? (3) At what price would you still find this product appealing yet expensive (expensive)? (4) What price is too high for the perceived quality and thus would prevent you from buying it (too expensive)? Systematic analysis of responses to the 4 key pricing judgments allowed us to identify product perception frequencies across price points and to compute their cumulative percentages.

Third, we calculated key pricing metrics—including the highest pricing point (HPP), acceptable price point (APP), optimal price point (OPP), and lowest pricing point (LPP), along with the acceptable price range (APR) for the HPV vaccines (24).

Finally, according to the van Westendorp price sensitivity meter (PSM) (24), we evaluated the difference between the OPP and APP for each vaccine as a measure of price sensitivity.

### 2.3 Statistical analysis

Survey data were collected via the “Wenjuanxing” platform (see Footnote 1), which provides direct export functionality to SPSS Statistics Data Files (.sav format). After the exclusion, invalid samples were removed according to the predefined exclusion criteria. The curated dataset was subsequently analyzed via SPSS version 28.0 (IBM Corporation, Armonk, NY, USA). Continuous variables are presented as the means ± SDs, and categorical variables are presented as *n* (%). Univariable analyses were used to assess associations between variables (demographics, HPV knowledge, HL, and various dimensions of VHS) and college students’ WTP for the HPV vaccine. For univariable analyses, continuous variables were compared via independent samples t tests or analysis of variance (ANOVA); categorical variables were analyzed via Pearson’s chi-square tests; for nonnormally distributed data, Mann–Whitney U tests or Kruskal–Wallis tests were applied. The variables with *p* < 0.05 in the univariable analyses were entered into a binary logistic regression model to identify independent predictors of college students’ WTP for the HPV vaccine. Adjusted odds ratios (ORs) with 95% confidence intervals (CIs) were computed, with statistical significance set at *p* < 0.05. Finally, Nagelkerke’s *R*^2^ was used to assess the goodness-of-fit of the binary logistic regression model.

## 3 Results

### 3.1 Sociodemographic characteristics

In this study, 66.9% (*n* = 3,297) of the participants were female and 33.1% (*n* = 1,631) were male college students. The mean age of the respondents was 20.69 ± 1.78 years. Among them, 55.2% (*n* = 2,721) resided in rural areas, 74.0% (*n* = 3,666) were medical-related majors, and 66.2% (*n* = 3,266) were enrolled in government health insurance programs. Additionally, 77.8% (*n* = 3,835) reported an average monthly consumption between 1,000 and 2,000 CNY (i.e., $140.65–$281.29, exchange rate benchmark date: June 16, 2024, converted at 1 USD = 7.11 CNY, hereinafter the same). Awareness of HPV was notably high, with 97.6% (*n* = 4,814) of participants reporting familiarity with the virus.

Furthermore, 78.2% (*n* = 3,852) expressed a willingness to receive the HPV vaccine, whereas 28.8% (*n* = 1,344) had either been vaccinated or had scheduled an appointment for vaccination (Table 1).

**Table 1:** Sociodemographic characteristics and univariable associations with the WTP for the HPV vaccine among college students.

Variable	Total, *N* (%)	Willingness to pay for the HPV vaccine out of pocket, *N* (%)		*p*
		No	Yes	
Socio-demographic characteristics				
Sex				**<0.001** ^**a**^
Female	3,297 (66.9)	762 (47.7)	2,535 (76.1)	
Male	1,631 (33.1)	837 (52.3)	794 (23.9)	
Age (M ± SD)	20.69 ± 1.78	20.67 ± 1.766	20.7 ± 1.782	0.55^b^
Education level				0.096^a^
Undergraduate	4,796 (97.3)	1,565 (97.9)	3,231 (97.1)	
Postgraduate	132 (2.7)	34 (2.1)	98 (2.9)	
Household registration				**<0.001** ^**a**^
Rural	2,721 (55.2)	939 (58.7)	1782 (53.5)	
Cities and towns	2,207 (44.8)	660 (41.3)	1,547 (46.5)	
Major				0.6^a^
Medical-related majors	3,666 (74.0)	1,182 (73.9)	2,484 (74.6)	
Nonmedical majors	1,262 (26.0)	417 (26.1)	845 (25.4)	
Monthly consumption (CNY)				**<0.001** ^**c**^
< 1,000 ($140.65)	403 (8.2)	163 (10.2)	240 (7.2)	
1,000–1,500 ($140.65–$210.97)	2,380 (48.3)	847 (53.0)	1,533 (46.0)	
1,501–2000 ($211.11–$281.29)	1,455 (29.5)	407 (25.4)	1,048 (31.5)	
2001–3,000 ($281.43–$421.94)	515 (10.5)	134 (8.4)	381 (11.4)	
>3,000 ($421.94)	175 (3.6)	48 (3.0)	127 (3.8)	
Government medical insurance participation				**<0.001** ^**a**^
No	1,662 (33.8)	624 (39.0)	1,038 (31.2)	
Yes	3,266 (66.2)	975 (61.0)	2,291 (68.8)	
Monthly household income (CNY)				**<0.001** ^**c**^
<1,500 ($210.97)	122 (2.5)	50 (3.1)	72 (2.2)	
1,500–2,500 ($210.97–$351.62)	293 (5.9)	112 (7.0)	181 (5.4)	
2,501–4,000($351.76–$562.59)	738 (15.0)	253 (15.8)	485 (14.6)	
4,001–8,000($562.73–$1125.18)	1,673 (33.9)	554 (34.6)	1,119 (33.6)	
>8,000($1125.18)	2,102 (42.7)	630 (39.4)	1,472 (44.2)	
Have you ever heard of HPV?				**<0.001** ^**a**^
No	114 (13.0)	80 (5.0)	34 (1.0)	
Yes	4,814 (87.0)	1,519 (95.0)	3,295 (99.0)	
Have you received or reserved the HPV vaccine?				**<0.001** ^**a**^
No	3,470 (71.2)	1,454 (95.7)	2016 (61.2)	
Yes	1,344 (28.8)	65 (4.3)	1,279 (38.8)	
Willingness to accept HPV vaccine				**<0.001** ^**a**^
No	1,076 (21.8)	646 (40.4)	430 (12.9)	
Yes	3,852 (78.2)	953 (59.6)	2,899 (87.1)	
HPV knowledge score (M ± SD)	12.44 ± 2.95	11.8 ± 3.121	12.75 ± 2.81	**<0.001** ^**b**^
eHealth literacy score (M ± SD)	31.65 ± 5.12	30.84 ± 5.338	32.04 ± 4.973	**<0.001** ^**b**^
HPV vaccine hesitancy scale (M ± SD)				
Necessity	7.25 ± 3.16	8.40 ± 3.29	6.70 ± 2.94	**<0.001** ^**b**^
Importance	20.69 ± 3.32	19.85 ± 3.32	21.10 ± 3.24	**<0.001** ^**b**^
Safety	10.54 ± 2.58	10.97 ± 2.34	10.34 ± 2.65	**<0.001** ^**b**^

a *p* value from the Pearson chi-square test.

b *p* value from the Mann–Whitney test.

c *p* value from the Kruskal–Wallis test.

Boldface indicates statistical significance (*p* < 0.05).

### 3.2 HPV knowledge and eHealth literacy

College students demonstrated marginally suboptimal HPV knowledge (mean = 12.44 ± 2.95 vs. passing the threshold of 12.8) and high eHL levels (overall mean = 31.65 ± 5.12; item-level mean = 3.96 > 3–a cutoff value) (Table 1).

### 3.3 Vaccine hesitancy

VHS analysis revealed distinct characteristics across dimensions: the necessity dimension demonstrated a mean score of 7.25 ± 3.16, which was below the critical threshold of 12. The importance dimension had significantly higher scores (20.69 ± 3.32), exceeding the critical threshold of 15. Notably, the safety dimension had a mean score of 10.54 ± 2.58, which was higher than the critical threshold of 9 (Table 1).

### 3.4 Willingness-to-pay

**Figure 1:**
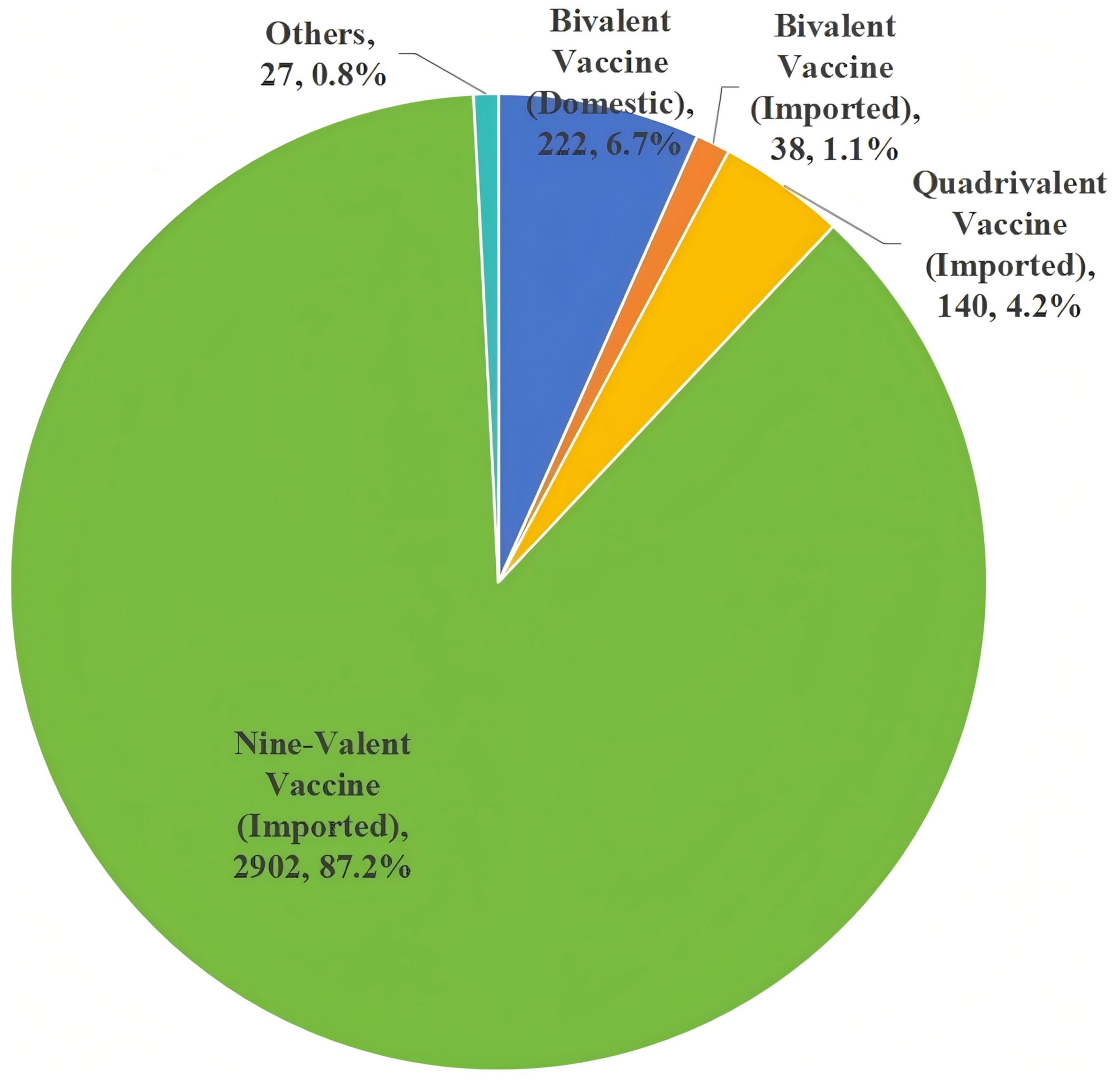
HPV vaccine preferences among college students.

This survey revealed that 67.6% (*n* = 3,329) of the college students were willing to pay for the HPV vaccine out of pocket. Among them, 87.2% (*n* = 2,902) preferred to receive the nine-valent HPV vaccine (imported) (Figure 1).

#### 3.4.1 Univariable analyses

The results of the univariable analyses assessing the associations between sociodemographic factors, HPV knowledge, HL, VH, and vaccine WTP are shown in Table 1.

Significant associations emerged between sex and household registration, monthly consumption, participation in government medical insurance, household income, HPV awareness, willingness to receive the HPV vaccine, and vaccination status (all *p* < 0.001).

The analyses also revealed that college students’ WTP for the HPV vaccine was associated with greater knowledge and HL and more favorable necessity, importance and safety scores for the VHS (all *p* < 0.001).

#### 3.4.2 Factors associated with the WTP for the HPV vaccine

Significant univariable predictors were entered into binary logistic regression (Table 2).

**Table 2:** Binary logistic regression for the WTP for the HPV vaccine among college students.

Variable	Logit coefficient	OR (95% CI)	*p*
Socio-demographic characteristics			
Sex			
Female	Ref		
Male	−1.19	0.31 (0.27–0.35)	**<0.001**
Household registration			
Rural	Ref		
Cities and towns	0.00	1 (0.87–1.14)	0.96
Monthly consumption (CNY)			**<0.001**
< 1,000 ($140.65)	Ref		
1,000–1,500 ($140.65–$210.97)	0.15	1.16 (0.91–1.48)	0.221
1,501–2000 ($211.11–$281.29)	0.42	1.52 (1.17–1.98)	**0.002**
2001–3,000 ($281.43–$421.94)	0.56	1.75 (1.27–2.42)	**<0.001**
>3,000 ($421.94)	0.58	1.79 (1.15–2.78)	**0.01**
Government medical insurance participation			
No	Ref		
Yes	0.32	1.38 (1.2–1.58)	**<0.001**
Monthly household income (CNY)			0.82
<1,500 ($210.97)	Ref		
1,500–2,500 ($210.97–$351.62)	−0.18	0.84 (0.52–1.34)	0.459
2,501–4,000 ($351.76–$562.59)	−0.02	0.98 (0.63–1.51)	0.913
4,001–8,000 ($562.73–$1125.18)	−0.07	0.94 (0.62–1.42)	0.762
>8,000 ($1125.18)	−0.01	0.99 (0.65–1.51)	0.95
HPV knowledge score	0.07	1.08 (1.05–1.1)	**<0.001**
eHealth literacy score	0.02	1.02 (1–1.03)	**0.013**
HPV vaccine hesitancy scale			
Necessity	−0.10	0.9 (0.89–0.92)	**<0.001**
Importance	0.07	1.07 (1.05–1.1)	**<0.001**
Safety	−0.09	0.91 (0.89–0.94)	**<0.001**

Boldface indicates statistical significance (*p* < 0.05). Nagelkerke’s *R*^2^ = 0.213, chi-square = 14.82, df = 8, *p* value = 0.063.

For VHS dimensions: OR <1 for perceived necessity and safety (reverse-scored dimensions) indicates lower perceived need/safety (i.e., higher disagreement/concern) predicts higher WTP. OR >1 for perceived importance (positively scored) indicates higher perceived importance predicts higher WTP.

The key predictors of the WTP for the HPV vaccine included sex, monthly consumption, and medical insurance participation. Compared with female college students, male college students presented a lower WTP (OR: 0.31; 95% CI: 0.27–0.35; *p* < 0.001). Higher monthly consumption was dose-responsively associated with greater WTP: >3,000 CNY vs. <1,000 CNY (OR = 1.79; 95% CI: 1.15–2.78; *p* = 0.01); 2001–3,000 CNY vs. <1,000 CNY (OR = 1.75; 95% CI: 1.27–2.42; *p* < 0.001); and 1,501–2000 CNY vs. <1,000 CNY (OR = 1.52; 95% CI: 1.17–1.98; *p* = 0.002). Medical insurance participation increased WTP (OR: 1.38; 95% CI: 1.20–1.58; *p* < 0.001).

Moreover, participants’ knowledge of HPV and HL positively influenced their WTP (OR: 1.08; 95% CI: 1.05–1.10; *p* < 0.001 and OR: 1.02; 95% CI: 1.00–1.03; *p* = 0.013, respectively).

Furthermore, for VHS, lower perceived necessity (i.e., higher reverse-scored disagreement on vaccine necessity; OR: 0.90; 95% CI: 0.89–0.92; *p* < 0.001), higher perceived importance (OR: 1.07; 95% CI: 1.05–1.10; *p* < 0.001) and lower perceived safety (i.e., higher reverse-scored safety concern; OR: 0.91; 95% CI: 0.89–0.94; *p* < 0.001) significantly predicted greater WTP (Table 2).

### 3.5 Van Westendorp PSM

Table 3 reveals notable differences in college students’ WTP and APRs (calculated between the lowest and highest price points) for various HPV vaccines. Specifically, the APRs (in CNY) identified for the bivalent HPV vaccine (domestic), bivalent HPV vaccine (imported), quadrivalent HPV vaccine (imported), and nine-valent HPV vaccine (imported) were [308.75, 459.72] [i.e., ($43.42, $64.66)], [510, 683.33] [i.e., ($71.73, $96.11)], [804.81, 995] [i.e., ($113.19, $139.94)], and [942.42, 1204.8] [i.e., ($132.55, $169.46)], respectively. The OPPs (equivalent ‘too cheap/too expensive’ perception proportions at this price) of these HPV vaccines were 376.67 CNY ($52.98), 610.00 CNY ($85.79), 877.50 CNY ($123.42), and 1061.19 CNY ($149.25), respectively. Notably, the market price of the nine-valent HPV vaccine (imported) was 1331.00 CNY (i.e., $187.20), which exceeds the APR for the college student population, whereas the market prices of the other vaccines fall within the acceptable range.

**Table 3:** van Westendorp price points (in CNY).

Price point	Bivalent HPV vaccine (domestic)	Bivalent HPV vaccine (imported)	Quadrivalent HPV vaccine (imported)	Nine-valent HPV vaccine (imported)
Highest pricing point (HPP)	459.72	683.33	995.00	1204.80
Acceptable price point (APP)	391.57	588.89	903.66	1069.10
Optimal price point (OPP)	376.67	610.00	877.50	1061.19
Lowest pricing point (LPP)	308.75	510.00	804.81	942.42
Market price	360	611	831	1,331
Acceptable price range (APR)	[308.75, 459.72]	[510, 683.33]	[804.81, 995]	[942.42, 1204.8]

**Figure 2:**
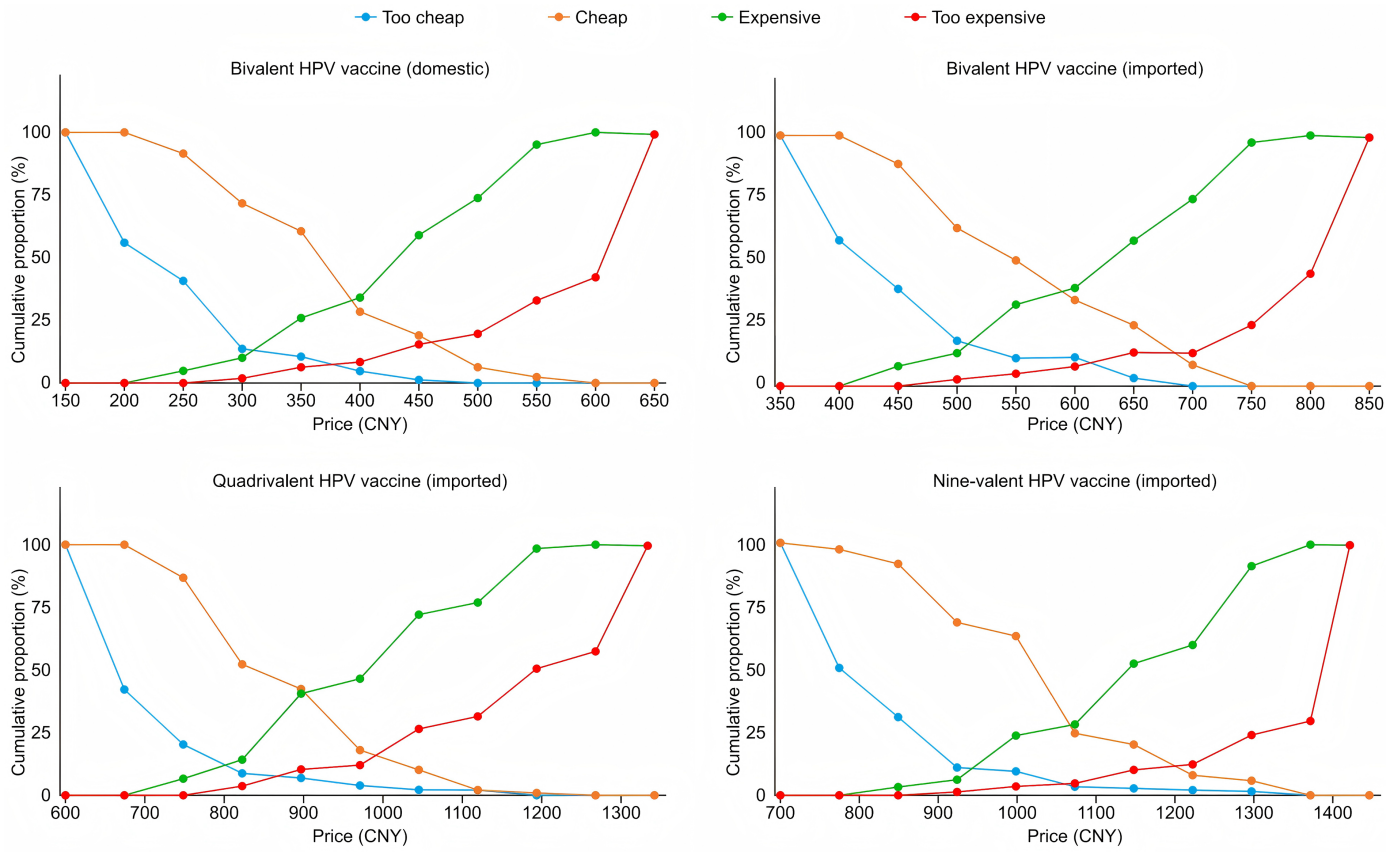
van Westendorp price sensitivity graphs for the HPV vaccines.

Additionally, to more clearly understand male college students’ price sensitivity toward different HPV vaccines, 4 cumulative response curves were generated from the data. Plots of the data for the van Westendorp PSM are presented in Figure 2.

For example, for the nine-valent HPV vaccine (imported), the APR (in CNY) was [942.42, 1204.8] [i.e., ($132.55, $169.46)]; that is, while consumers would consider a price higher than 1204.8 CNY ($169.46) to be too expensive, a price lower than 942.42 CNY ($132.55) would likewise imply considerable loss of potential consumers owing to their concerns about the quality of the product. The current market price of 1,331 CNY represents a 126.2 CNY ($17.75) premium over the maximum affordable price threshold for college students (1,204.8 CNY), demonstrating a significant affordability barrier. Furthermore, we found that the OPP (the intersection of the “too cheap” and “too expensive” curves) was approximately 3.5%, which means that 96.5% of the participants believe that when the price is approximately 1061.19 CNY ($149.25), it is neither too expensive nor too cheap. Moreover, at the OPP, provider sustainability and universal coverage requirements are balanced. The current market price exceeds the balance point by 269.81 CNY ($37.95), demonstrating a significant price premium. The APP (the intersection of the “cheap” and “expensive” curves) was 1069.1 CNY ($150.37), indicating that at this price point, the number of consumers who perceive the price is slightly equal to the number who view it as good value (Figure 2).

Notably, with respect to price sensitivity, customers exhibit heightened responsiveness to price variations when the APP and OPP of a product converge. This observation revealed that the nine-valent vaccine (imported) demonstrated the highest sensitivity among the four comparable HPV vaccine products.

## 4 Discussion

### 4.1 Willingness to pay for the HPV vaccine

This study reveals a critical paradox in HPV vaccine uptake among Chinese college students. Despite a high willingness to receive (78.2%) and pay for the HPV vaccine (67.6% willing to self-fund), the actual vaccination rate remains notably low (28.8%), underscoring a substantial gap between intention and action. This observed intention–behavior gap is a hallmark of vaccine hesitancy, a complex and globally recognized phenomenon (25, 26). These findings are consistent with previous studies in China reporting similar disparities in HPV vaccine acceptance versus actual uptake (10, 27–29). Notably, while the magnitude of the gap in our study aligns with national observations, the specific barriers contributing to hesitancy both domestically and internationally (e.g., safety concerns, cost issues, knowledge gaps) need to be analyzed to understand both universal factors and context-specific factors (30, 31). Therefore, there is an urgent need for evidence-based targeted interventions specifically addressing the drivers of HPV vaccine hesitancy to address the persistent disparities between intentions and vaccinations and to increase HPV vaccination rates among college students.

### 4.2 Sociodemographic characteristics

This study revealed that as consumption levels increase, college students’ WTP for HPV vaccines also gradually increases, which aligns with the results of existing research (10, 32). International comparisons revealed similar patterns; studies in Bangladesh and Thailand revealed that HPV vaccine acceptance was significantly positively correlated with income levels (33, 34), whereas young people with higher socioeconomic status in Switzerland also demonstrated greater vaccination uptake (35). Even the relatively low-priced domestic bivalent HPV vaccine represents a considerable expense for college students, however, especially compared with middle- and high-income countries (36). When the vaccine price is within a reasonable range, both the vaccination rate and WTP for the HPV vaccine tend to be relatively high (18), as evidenced by the 90% vaccination rate among countries such as Portugal schoolgirls benefiting from free national immunization programs (36), which contrasts with China’s 14.02% first-dose coverage at most (7). Therefore, policy interventions aimed at reducing vaccine prices may be effective, as demonstrated by the successful implementation of free vaccination programs in 115 countries (36).

Additionally, this study revealed a greater WTP for the HPV vaccine among government-insured participants, which aligns with existing research in China (37) and international findings showing that health insurance in the U.S. greatly increased the likelihood of being vaccinated against HPV (38). Existing evidence shows that insurance enrollment reduces VH-related behaviors by improving perceived necessity and lowering financial barriers (39). Integrating HPV vaccines into medical insurance and prioritizing student enrollment could thus increase uptake through cost reduction and preventive health reinforcement.

### 4.3 Health literacy and HPV knowledge

The results of this study confirm that HL and HPV knowledge positively predict WTP for HPV vaccination. This finding aligns with several national and international studies reporting similar positive associations between HL, vaccine knowledge, and willingness to accept various vaccinations, including HPV (40–42). Research has shown that HL is associated with healthier behaviors and vaccination uptake (43, 44). Additionally, students with greater HPV knowledge demonstrated higher HL levels (45). Greater HL and HPV knowledge improves the understanding of the benefits and risks of the HPV vaccine, reduces misconceptions and vaccine hesitancy, and increases the WTP for preventative strategies. Targeted education that improves HPV knowledge simultaneously improves HL, suggesting that dual-focused interventions could effectively increase vaccine acceptance rates.

### 4.4 Vaccine hesitancy

The study revealed that the perceived necessity for HPV vaccination most significantly influenced college students’ WTP, which aligns with previous studies’ findings (9, 39, 46). Notably, this phenomenon mirrors vaccine hesitancy patterns observed among Chinese guardians of adolescents, where 21.1% delayed vaccination due to uncertainty about necessity (22). In this study, while most participants recognized its importance, those dismissing vaccination due to a perception of strong immunity or good health or a lack of government mandate showed a reduced WTP. Cross-nationally, European studies indicate that “complacency” (e.g., low risk perception) contributes to 38% of hesitant individuals maintaining refusal status over time, particularly in populations with high education levels (47). Campus education should emphasize that the risk of HPV infection is not determined by individual health and should promote the value of vaccination despite the absence of mandatory programs.

The importance dimension significantly and positively influenced college students’ WTP for the HPV vaccine, which aligns with the findings of previous studies (10). The central role of perceived importance in driving WTP resonates with broader international findings on vaccine confidence frameworks, particularly the WHO’s Behavioral and Social Drivers (BeSD) model, where belief in a vaccine’s effectiveness, personal relevance, and perceived importance serve as critical global determinants influencing vaccination decisions (48). Specifically, college students who perceived that the HPV vaccine effectively prevents diseases such as cervical cancer and improves both their own health and their family’s health reported a greater WTP. Within the domestic context, studies among guardians of adolescents also highlight that recognizing the importance of vaccines significantly reduces hesitancy (22). Promoting awareness of the personal and societal benefits of the vaccine remains crucial.

College students’ concerns about the safety of the HPV vaccine negatively impacted their WTP. Despite robust evidence supporting the safety and efficacy of the vaccine, public apprehension remains prevalent (22, 49, 50). Safety concerns are the primary factor contributing to vaccine hesitancy, which is not only prevalent among this student population but has also been widely reported in domestic studies (e.g., concerns about side effects remain a major barrier) (28, 51, 52) and is internationally recognized as one of the key pillars of the “3C” model of vaccine hesitancy (complacency, convenience, confidence) (50, 53). Increasing trust in vaccine safety and healthcare expertise is vital for improving vaccination rates.

### 4.5 van Westendorp PSM

van Westendorp PSM analysis revealed that while the market prices of other vaccine types generally aligned with the APRs, multiple studies internationally and within China have confirmed that the nine-valent vaccine consistently exceeds both optimal pricing thresholds and affordability limits for consumers, including college students (19, 54, 55). Consistent with patterns observed in other countries (56), comparative surveys in China have revealed that domestic vaccines maintained lower acceptable prices (CNY 910.63–2,866.96) than their imported counterparts did (CNY 1,689.80–3,252.43) (57). Notably, a 2021 national survey of healthcare workers reported median willingness-to-pay values (CNY 1,250–1,400) that were below the prevailing market prices (58). Although historical price acceptability gaps have narrowed for most HPV vaccines since 2018 (27), mirroring trends seen elsewhere as vaccine markets mature (59), the nine-valent vaccine continues to demonstrate persistent market price resistance in the Chinese context. This suggests sustained consumer sensitivity to its premium pricing strategy and that appropriate price reductions may significantly boost the uptake of the nine-valent vaccine.

The van Westendorp PSM effectively quantified HPV vaccine price sensitivity and consumer value perceptions, enabling the development of precise pricing strategies for policymakers. To increase accessibility, government interventions should prioritize cost-optimized subsidies to integrate the nine-valent HPV vaccine into medical insurance schemes, thereby mitigating persistent affordability disparities. According to the results of our econometric analysis, we propose a tiered subsidy framework targeting college students: (1) a compensation of 269.81 CNY ($37.95) to achieve near-OPP thresholds or (2) a minimum subsidy of 126.20 CNY ($17.75) ensuring expenditure alignment with the APRs derived from the van Westendorp PSM.

## 5 Limitations

This study has several limitations. First, its sampling scope—restricted to one medical university—may introduce regional and educational biases and thus limit the generalizability of our findings to broader socioeconomic groups. Second, the use of a self-reported WTP risks inducing social desirability bias, potentially inflating the stated purchase intentions. Third, the small sample sizes for the non-9-valent vaccines and the cross-sectional design limit the development of causal inferences. Future research should validate these findings through multisite sampling, real-world purchase tracking, and WTP assessments across diverse demographic and economic contexts.

## 6 Conclusion

This study revealed a high WTP and a strong preference for the nine-valent HPV vaccine. WTP was shaped by HL and VH (particularly in terms of perceptions of vaccine necessity, importance, and safety) alongside socioeconomic factors such as monthly consumption, medical insurance, and HPV knowledge. The van Westendorp PSM confirmed an acute price sensitivity for the imported nine-valent vaccine, and its market price exceeds college students’ affordability thresholds. To address these barriers, we recommend prioritizing a dual intervention: (1) Affordability reforms, such as integrating HPV vaccines into medical insurance and implementing of targeted tiered subsidies (e.g., an optimal subsidy of 269.81 CNY, or at least 126.20 CNY) of the nine-valent HPV vaccine for low-income students and incentivizing local production to reduce costs; and (2) behavioral “nudges,” including the tailoring of campus education to simultaneously improve HL and counter VH by emphasizing the universal risks of HPV and the need for and safety evidence of HPV vaccines. Multisectoral strategies aligning pricing adjustments (guided by PSM data), targeted subsidies, and theory-driven education are critical to convert intent into action.

## Data Availability

The datasets presented in this article are not readily available because of personal privacy and ethical restrictions. Requests to access the datasets should be directed to HK, khiromi@hiroshima-u.ac.jp.

## Footnotes

^1^ www.wjx.cn
